# Guidance of adjuvant instillation in intermediate-risk non-muscle invasive bladder cancer by drug screens in patient derived organoids: a single center, open-label, phase II trial

**DOI:** 10.1186/s12894-023-01262-1

**Published:** 2023-05-11

**Authors:** Roland Seiler, Martin Egger, Marta De Menna, Saskia Wehrli, Martina Minoli, Martina Radić, Pavel Lyatoshinsky, Raphael Hösli, Jennifer Blarer, Dominik Abt, Marianna Kruithof-de Julio

**Affiliations:** 1 Department of Urology, Hospital Center Biel, Spitalzentrum Biel, Vogelsang 84, 2501 Biel, Switzerland; 2 Department for BioMedical Research, Translational Organoid Resource Core, University of Bern, Bern, Switzerland; 3 Department of Urology, St. Gallen Cantonal Hospital, St. Gallen, Switzerland; 4 Department of Pharmacy, Hospital Center Biel, Biel, Switzerland; 5 Department for BioMedical Research, Urology Research Laboratory, University of Bern, 3008 Bern, Switzerland; 6 Department of Urology, Inselspital, Bern University Hospital, 3010 Bern, Switzerland

**Keywords:** Non-muscle invasive bladder cancer, Patient derived organoids, Precision oncology, Adjuvant instillation therapy

## Abstract

**Background:**

In intermediate-risk non-muscle invasive bladder cancer (NMIBC) clinical guidelines suggest an adjuvant instillation with a chemotherapeutic agent. However, the agent and regimen are not clearly defined. Worldwide, less than 15% of patients receive this adjuvant chemotherapeutic instillation. We recently developed a pipeline for the generation of patient derived organoids (PDO) in NMIBC. In this phase II trial, we aim to use our in vitro pipeline to select the most effective drug for chemotherapeutic instillation in NMIBC patients.

**Methods:**

Patients with first diagnosis of intermediate-risk NMIBC that are directed to transurethral resection of bladder tumor (TURBT) are enrolled. During TURBT, tumor is sampled, and specimens are directed to generate PDO. Once the PDO are formed, drug screens on them for Epirubicin, Mitomycin C, Gemcitabine and Docetaxel are performed. The drug with the highest antitumor activity in vitro will then be selected for 6 adjuvant intravesical instillations once weekly. Thereafter, patients are followed according to clinical guidelines by cystoscopy.

**Discussion:**

The aim of this trial is to use drug screens in PDO to precise treatment selection for adjuvant instillation therapies in patients with intermediate-risk NMIBC. The ultimate goal of this trial is to reduce the risk of cancer recurrence. In the future, we aim to conduct clinical multicenter trials with an increased sample size, a broader panel of compounds and a focus on the reduction of cancer recurrence by precision delivery of care.

*Trial registration* NCT05024734.

## Background

Bladder cancer is one of the most common cancers in the Western World and the second most common genitourinary malignancy [1]. Due to its high incidence, bladder cancer results in a high associated morbidity and mortality [2]. Even though only 25% of patients present with a muscle invasive bladder cancer, the high recurrence and progression rates of up to 60% in ten years makes non-muscle invasive bladder cancer (NMIBC) the costliest cancer to treat on a per patient basis. This is mostly due to the related tumor follow-up [3].

Prior to the final histological diagnosis during a transurethral resection of the bladder tumor (TURBT) patients undergo a cystoscopy and cytological analysis. Depending on the macroscopical results during cystoscopy and TURBT (invasion, tumor size, single/multiple), as well as the histological diagnosis (high grade/low grade) and the age of the patient, bladder cancer is stratified into low-, intermediate-, high- and very-high risk groups [1]. All patients with NMIBC should undergo an early chemotherapeutical instillation as it is considered to reduce reimplantation of cancer cells and reduce the recurrence risk [1].

Patients with low-risk disease undergo an early single adjuvant instillation of a chemotherapeutical agent [4]. For those with a high-grade bladder cancer, a second TURBT to confirm the complete resection of the tumor is recommended, followed by an induction and maintenance cycle of intravesical Bacillus Calmette-Guérin (BCG) within 6–7 weeks after diagnosis to elicit an immune response as an adjuvant treatment [1].

Early instillation, induction cycle and maintenance for one year of chemotherapy into the bladder is recommended for intermediate-risk NMIBC by EAU guidelines. Despite the optimal schedule and duration of further intravesical chemotherapy instillation is not defined, it should not exceed one year [1]. As surveys show, low-risk bladder cancer patients seem to be overmonitored and those with high-risk undermonitored. In addition, only 37% of intermediate-risk patients receive an extended instillation chemotherapy [5].

There is no specific regimen for intravesical chemotherapy defined by the recent EAU-Guidelines [1]. Best practice shows that adjuvant treatment is started with an induction cycle of 6 intravesical instillations [6]. The most frequently used agents for intravesical chemotherapy are Mitomycin C (MMC) and Epirubicin. Results with newer drugs are emerging, but long-term follow-up data are still lacking [7]. Gemcitabine and Docetaxel seem to be two very promising agents for further investigation. Gemcitabine shows remarkable response rates even in BCG refractory patients [8]. Response rates for Docetaxel are around 56% in early phase trials [9].

Until recently, the treatment of bladder cancer, for several years, was limited to surgery and to immunotherapy or chemotherapy. Currently, the extensive analysis of molecular alterations has led to novel treatment approaches [10]. The molecular landscape of NMIBC is heterogeneous [11, 12]. Not only the mutational profile but also the transcriptomic characteristics vary between different NMIBC. Although different agents are used on a routine daily base and in clinical trials, they have not been administered based on the molecular landscape or biological likelihood of response.

Patient derived organoids (PDO) represent the molecular aspects of the primary tumor and can not only be used to understand the biological basis of the disease, but may also be used to develop new treatment strategies. Organoids are nowadays used for research of different cancers. In the field of urology, there are studies/reports on kidney, prostate, and bladder organoids [13].

Taken together, adjuvant intravesical chemotherapy is considered to reduce the recurrence risk of intermediate-risk bladder cancer. A clearly defined regime of therapy induction and maintenance is still lacking but should not exceed one year. The heterogeneity of non-muscle invasive bladder cancer is related to a high variability of efficacy of chemotherapeutic agents. Even though Epirubicin and MMC are the most frequently used drugs for adjuvant instillation therapy, Docetaxel and Gemcitabine show promising results in existing studies. In vitro drug screens in PDO may predict the response of the agent in vivo, but the implementation of in vitro drug screens in PDO in clinical settings are still pending.

## Methods/design

### Study aim and objectives

The aim of this trial is to test the possibility of using drug screens in PDO to guide intravesical instillation.

#### Overall objective

Currently, the drug for chemotherapeutic instillation after TURBT in patients with intermediate-risk low grade NMIBC is selected based on doctors’- and institutional preferences. Most frequently, Epirubicin or MMC are used. Currently, no comparative studies focus on this patient population between the different drugs and the selection is not based on biological characteristics.

We aim to establish a workflow into the clinical routine, that allows a specific selection of the instilled drug based on molecular characteristics of the respective NMIBC.

The current study will test the use of drug screens in PDO for the prediction of treatment in patients. Therefore, we will test the implementation of drug screen in PDO in the workflow of the daily routine.

#### Primary objective

The primary objective of this study is to evaluate the rate of successful drug selection by using drug screens in PDO generated from patients with low grade intermediate-risk NMIBC. This will allow to determine the rate of patients in which drug selection can successfully be performed for adjuvant intravesical instillation therapies.

#### Secondary objectives

Secondary objectives of the trial are to evaluate the rate of recurrence, the recurrence free survival and the progression free survival in this population. Further quality of life and safety of instillation will be assessed.

### Ethical approval and consent

The GAIN-INST Trial (NCT05024734) has been approved by swissethics and the district ethical board from Bern (BASEC ID 2021-02369). All included patients have read the study information and signed the informed consent for this trial.

### Enrollment and trial design

**Fig. 1:**
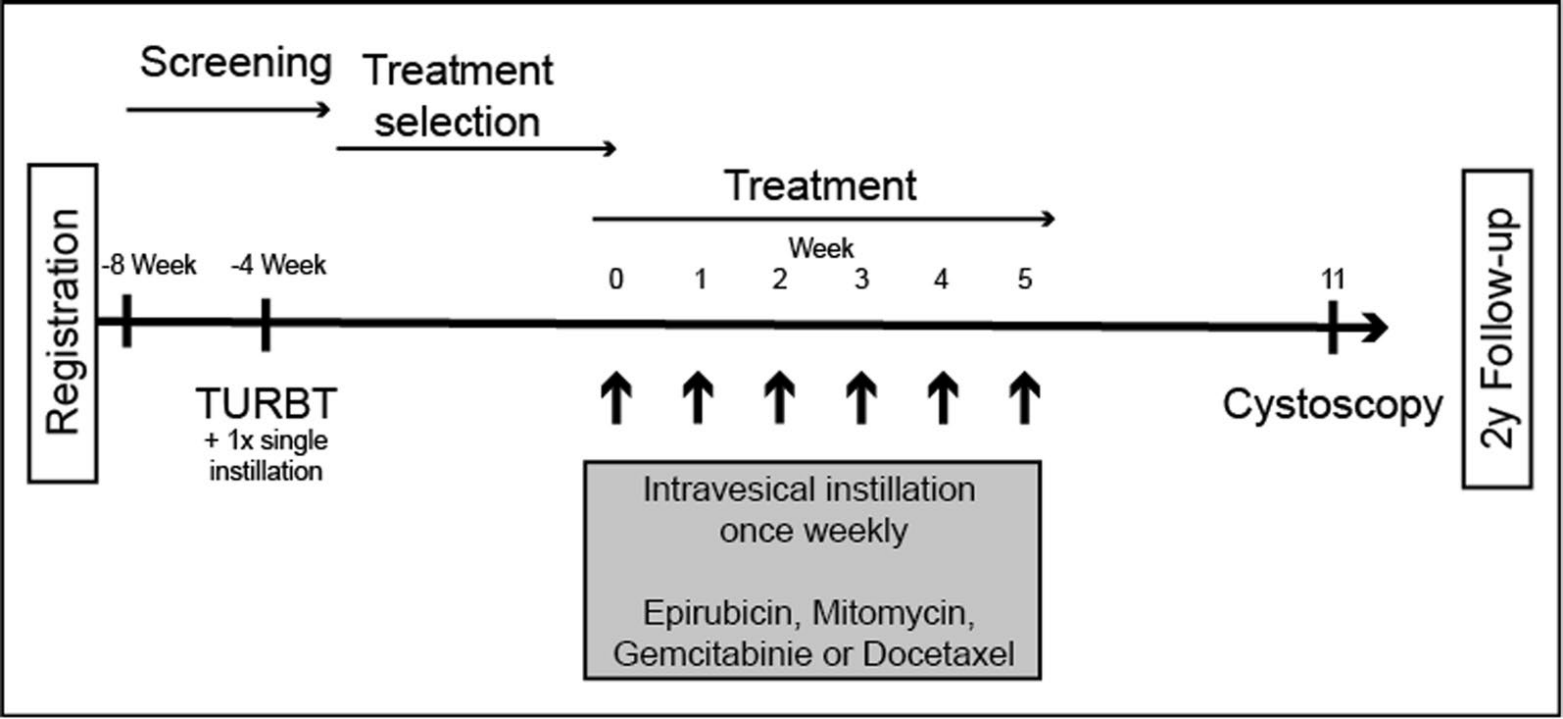
Trial design

The Department of Urology at the Hospital Center in Biel is conducting the GAIN-INST Trial in which from September 2022 for two years, patients with intermediate-risk NMIBC are recruited according to the criteria in Table 1. After signing the informed consent, patients will be included. In this clinical single-center phase II trial, patients with primary diagnosis of NMIBC are screened in the outpatient clinic for trial recruitment prior TURBT. Tissue harvested during TURBT is used to generate PDO and perform drug screens. Four weeks after TURBT the identified drug is instilled intravesically once a week for 6 weeks. Thereafter, patients are followed according to the standard of care by cystoscopy (Fig. 1). The study is completed after a follow-up of two years, while clinical follow-up will continue to at least 5 years, respectively.

**Table 1:** Inclusion and exclusion criteria

Eligibility criteria	
Subjects fulfilling all of the following *inclusion* criteria are eligible for the study:	
1	Informed consent as documented by signature
2	Age ≥ 18 years
3	ECOG performance status of 0 or 1
4	Histologically confirmed intermediate-risk non-muscle invasive urothelial carcinoma of the bladder (pTa low grade)
5	Representative fresh tumor specimen for PDO generation and drug screen available.The presence of representative fresh tumor tissue will be confirmed as a re-assessment of eligibility at time point of surgery (Visit 2)
The presence of any one of the following *exclusion* criteria will lead to exclusion of the subject:	
1	Known previous high grade and/or high-risk non-muscle invasive bladder cancer
2	Previous intravesical biological/immune- (BCG) therapy
3	Evidence of significant uncontrolled concomitant disease that could affect compliance with the protocol as judged by the investigator
4	Severe infection within 4 weeks prior to cycle 1, day 1
5	Contraindication for frequent catheterization
6	Voiding dysfunction
7	Pregnancy or nursing
8	Female subject of childbearing potential who is unwilling to use acceptable method(s) of effective contraception during study treatment and through 6 months after the last treatment. Note: Women not of childbearing potential are defined as: postmenopausal (defined as at least 12 months with no menses without an alternative medical cause; in women < 45 years of age a high follicle stimulating hormone (FSH) level in the postmenopausal range may be used to confirm a post-menopausal state in women not using hormonal contraception or hormonal replacement therapy. In the absence of 12 months of amenorrhea, a single FSH measurement is insufficient.) OR have had a hysterectomy and/or bilateral oophorectomy, bilateral salpingectomy or bilateral tubal ligation/occlusion, at least 6 weeks prior to screening; OR has a congenital or acquired condition that prevents childbearing
9	Male subject who is unwilling to use acceptable method of effective contraception during IMP treatment and through 6 months after the last dose of IMP. For this trial, male subjects will be considered to be of non-reproductive potential if they have azoospermia (whether due to having had a vasectomy or due to an underlying medical condition)

### Sample size

The alternative hypothesis states that a successful drug prediction using drug screens in PDO is possible in more than 65% of cases. Sample size was determined using a one sample test. The null hypothesis that a drug prediction will only be possible in 65% of patients will be tested against a one-sided alternative (that the proportion is larger than 65%). Assuming that the real proportion is 85%, a sample of 31 participants should show a statistically significant result at 5% significance and 80% power using an exact binomial test. Assuming an early termination of 10% of patients, a total number of 34 patients will have to be included. Thus, the study will recruit 31 patients that have completed the full 6 instillation cycles.

### Tissue sampling, PDO generation and drug treatment

During surgery, cold cup biopsies will be harvested, digested to single cells and plated organoids are allowed to form. Thereafter, PDO are dissociated to single cells, plated and formed after 48 h. These PDO are then exposed to the compounds in the following conditions: untreated (medium) or treated (0.1% DMSO vehicle control, and 4 drugs that are currently used for intravesical instillation (Epirubicin, Mitomycin C, Gemcitabine and Docetaxel). Compound effect is then measured after 48 h by the CellTiterGlo 3D reagent from Promega measuring luminescense. For intravesical instillation, only these four drugs will be used. However, in-vitro testing will eventually include other compounds, dependent on the number of PDO available.

### Readout, drug selection and instillation

Each condition will be investigated in triplicates; triplicates and average will be used for drug selection. Only drugs that reduce viability by 50% or more compared to the vehicle, will be considered. In case that more than one drug reduces viability by 50% or more compared to the vehicle, a pairwise Wilcoxon test will be performed between drug and the vehicle. The drug with the lowest p-value will be selected.

Four weeks after TURBT, this selected drug will be used for intravesical instillation. Based on the results of the drug screen in PDO, patients will receive one of either (Epirubicin intravesical (50 mg/50 ml), Mitomycin C intravesical (40 mg/50 ml), Gemcitabine intravesical (1000 ml/50 ml) or Docetaxel intravesical (37.5 mg/50 ml)) once weekly for 6 weeks.

### Follow-up and data collection

The patients will be followed by cystoscopy according to the clinical guidelines [1]. The duration of follow-up as part of the clinical trial is done for 2 years after TURBT. However, our institutional guidelines for non-muscle invasive bladder cancer include regular follow-up visits after primary treatment until 5 years after TURBT.

The CRF will be electronic. All data requested on the eCRF must be recorded and the recorded data should be consistent with the source documents or the discrepancies should be explained. The Investigator ensures the accuracy, completeness, and timeliness of the data reported in the eCRF and all other required reports. Generally, the eCRF should be completed within one week of completion of a participant’s visit/ follow-up phone call.

The CRFs in this trial are implemented electronically using a dedicated electronic data capturing (EDC) system (REDCap ®, web-address: redcap.ctu.unibe.ch). The EDC system is activated for the trial only after successfully passing a formal test procedure. All data entered in the eCRF are stored on a Linux server in a dedicated Oracle database.

### Statistical methods for primary and secondary outcomes

The predictive potential of biomarkers for the primary and secondary outcome will be analysed using logistic regression.

Missing data is expected to occur due to drop-outs. The amount and reason of missing data will be reported for all outcomes. For survival outcomes drop-outs will be censored at the time of the drop-out.

### Data monitoring

For quality control of the study conduct and data retrieval, monitoring will be performed according to a monitoring plan determined by the Clinical Trial Unit from the University of Bern. Any findings and comments will be documented in site visit reports and communicated to the Sponsor-Investigator as applicable. Site staff will support the Monitor in his/ her activities. All source data and relevant documents will be accessible to Monitors and questions of Monitors are answered during site visits. All involved parties will keep participant data strictly confidential.

## Discussion

The major clinical challenges in NMIBC are the high rate of recurrence and the lack of a more precise delivery of care. Despite the molecular heterogeneity of NMIBC, all patients are treated according to the same protocol and followed by invasive and potentially harmful procedures.

Therefore, we aim to precise the treatment of NMIBC thus making the therapy more effective.

We believe that a more precise adjuvant treatment of NMIBC patients will reduce the risk of cancer recurrence and that drug screens in PDO may be a promising strategy to precise drug selection for intravesical instillation. The establishment of our protocols as part of clinical trials will allow us to investigate novel compounds for the intravesical treatment of NMIBC patients. Our in-vitro experiments may suggest other compounds for intravesical instillation that have not yet been investigated. In the future, we aim to conduct clinical multicenter trials with an increased sample size, a broader panel of compounds and a focus on the reduction of cancer recurrence by precision delivery of care.

NMIBC is the costliest cancer on a per patient treatment basis. All patients are treated in a “one size fits all” manner and the workup at diagnostics and during follow-up is invasive and may be harmful. The success of this project will help to precise the treatment of patients with intermediate-risk NMIBC. Further, the establishment of our pipeline, will allow an effective and biologically driven investigation of novel compounds for intravesical instillation therapies.

## Data Availability

The trial sponsor will have access to the final trial dataset, and disclosure of contractual agreements that limit such access for investigators.

## Abbreviations

AE: Adverse event
BASEC: Business administration system for ethical committees, (https://submissions.swissethics.ch/en/)
BCG: Bacillus Calmette-Guérin
CA: Competent authority (Swissmedic)
CEC: Competent ethics committee
CRF: Case report form
eCRF: Electronic case report form
CT: Computed tomography
CTCAE: Common terminology criteria for adverse events
DSUR: Development safety update report
EAU: European association of urology
ECOG: Eastern cooperative oncology group
IMP: Investigational medicinal product
ISO: International organisation for standardisation
ITT: Intention to treat
MMC: Mitomycin C
MRI: Magnetic resonance imaging
NCT: National clinical trial
NMIBC: Non-muscle invasive bladder cancer
PDO: Patient derived organoids
PI: Principal investigator
RBC: Red blood cells
SDV: Source data verification
SOP: Standard operating procedure
SPC: Summary of product characteristics
SUSAR: Suspected unexpected serious adverse reaction
TMF: Trial master file
TURBT: Transurethral resection of bladder tumor
WBC: White blood cells
